# P023. Reasons for headache investigation and findings in an experimental headache center

**DOI:** 10.1186/1129-2377-16-S1-A189

**Published:** 2015-09-28

**Authors:** Alessandro Panconesi, Maria L Bartolozzi, Leonello Guidi, Sandro Santini, Nedo Mennuti, Vincenzo Carini

**Affiliations:** Headache Center, Department of Neurology, Health Authority 11, Empoli, Italy; Radiodiagnostic Department, Health Authority 11, Empoli, Italy; Primary Care, Health Authority 11, Empoli, Italy; Radiodiagnostic Institute Ecomedica, Empoli, Italy

## Background

Warning symptoms or “red flags” are useful in targeting which patients with headache require investigation. Many red flags, even with normal neurological examination, are the cause of neuroimaging (CT or MRI) overutilization, in addition to patient reassurance. Optimizing headache neuroimaging practices should be a major priority. The aim of our study was to evaluate the investigation rate in patients referred for the first time in the period from 2011 to 2013 to our Headache Center (HC) conducted by a general practitioner particularly an expert in headache management, and to correlate the reasons of investigation with neuroradiological findings.

## Results

A total of 118 (10.9%) of 1,078 new patients (802 females, 276 males; mean age 41±15; range 7-90), 85% suffering from episodic or chronic migraine, were referred for neuroimaging: 107 MRI (20 MR angiography), 11 CT. Considering only the 676 subjects whom had never undergone neuroimaging, the percentage was 14.6. Sixteen out of 118 patients were investigated in the past (11 CT, 5 MRI).

The reasons for headache investigation were: recent change in characteristics (6), significant increased frequency from 1-12 months (55, in 21 daily headaches), recent (1-12 months) onset (25, in 14 daily headaches *ab initio* from 1-6 months), recent onset in patients over 40 years (19), abnormal neurological signs (12): alteration of Mingazzini or Romberg test, precipitated by exertion (8), atypical aura (8), first-degree relatives died from cerebral aneurism (4), memory deficit (4), migraine associated vertigo (7), paresthesia not typical of aura (7), nighttime onset (3), atypical cluster headache (1), trigeminal neuralgia first branch (1), recent thunderclap headache (1).

Twenty-two patients currently in good health had not performed the requested neuroimaging. Information regarding 9 residents outside the region was unavailable. The analysis of neuroimaging findings (82 MRI, 5 CT) therefore concerned 87 patients aged 14-78 years, 53 of them with migraine without aura and 11 with migraine with aura.

Insignificant abnormalities were found in 33 patients: paranasal sinus thickening (13), septum pellucidum cyst (2), pineal cyst (3), arachnoid cyst (3), circle of Willis variants (6), signs of chronic cerebral ischemia (5), doubtful small subependymoma (1). Significant abnormalities possibly related to headache were found in two patients (2.2%) with cavernous angioma and intracranial hypotension.

## Conclusions

The rate of headache patients investigated through neuroimaging was largely inferior to that previously reported in various clinical settings [1–3]. We suggest that a major study should evaluate if some red flags such as changes in headache characteristics but with normal neurological examination require investigation.

Written informed consent to publish was obtained from the patient(s).
